# The Impact of Social Media Use on Mental Health and Family Functioning Within Web-Based Communities in Saudi Arabia: Ethnographic Correlational Study

**DOI:** 10.2196/44923

**Published:** 2024-01-16

**Authors:** Bdour Alwuqaysi, Alfie Abdul-Rahman, Rita Borgo

**Affiliations:** 1 King's College London London United Kingdom

**Keywords:** social media use, mental health, family functioning

## Abstract

**Background:**

In recent years, increasing numbers of parents, activists, and decision-makers have raised concerns about the potential adverse effects of social media use on both mental health and family functioning. Although some studies have indicated associations between social media use and negative mental health outcomes, others have found no evidence of mental health harm.

**Objective:**

This correlation study investigated the interplay between social media use, mental health, and family functioning. Analyzing data from 314 users, this study explores diverse mental health outcomes. The study places particular emphasis on the Saudi Arabian sample, providing valuable insights into the cultural context and shedding light on the specific dynamics of social media’s impact on mental well-being and family dynamics in this demographic context.

**Methods:**

We collected data through a subsection of an anonymous web-based survey titled “The Effect of COVID-19 on Social Media Usage, Mental Health, and Family Functioning.” The survey was distributed through diverse web-based platforms in Saudi Arabia, emphasizing the Saudi sample. The participants indicated their social media accounts and estimated their daily use. Mental health was assessed using the General Health Questionnaire and family functioning was evaluated using the Family Assessment Device Questionnaire. In addition, 6 mental health conditions (anxiety, self-esteem, depression, body dysmorphia, social media addiction, and eating disorders) were self-reported by participants.

**Results:**

The study demonstrates a pattern of frequent social media use, with a significant portion dedicating 3-5 hours daily for web-based activities, and most of the sample accessed platforms multiple times a day. Despite concerns about social media addiction and perceived unhealthiness, participants cited staying connected with friends and family as their primary motivation for social media use. WhatsApp was perceived as the most positively impactful, whereas TikTok was considered the most negative for our Saudi sample. YouTube, Instagram, and Snapchat users reported poorer mental health compared with nonusers of these platforms. Mental health effects encompassed anxiety and addiction, with age and gender emerging as significant factors. Associations between social media use and family functioning were evident, with higher social media quartiles correlating with a greater likelihood of mental health and unhealthy family functioning. Logistic regression identified age and gender as factors linked to affected mental health, particularly noting that female participants aged 25-34 years were found to be more susceptible to affected mental health. In addition, multivariable analysis identified age and social media use quartiles as factors associated with poor family functioning.

**Conclusions:**

This study examined how social media affects mental health and family functioning in Saudi Arabia. These findings underscore the need for culturally tailored interventions to address these challenges, considering diverse demographic needs. Recognizing these nuances can guide the development of interventions to promote digital well-being, acknowledging the importance of familial connections in Saudi society.

## Introduction

Individual’s lives worldwide are now mostly impacted by social media [1]. By enabling individuals to retain offline connections and provide a more welcoming setting for emotional self-disclosure and help-seeking, social media might have a positive influence on their lives [2]. However, there have been rising worries about the possible detrimental impact of social media on individual mental health among certain scholars, governmental organizations, and the public [3]. Prior research has related social media use to multiple mental health conditions including body dissatisfaction, eating disorders, depressive symptoms, and social anxiety [4]. Facebook users, for instance, report higher body dissatisfaction than nonusers, according to a study involving male and female adults [5]. Another example of female adults revealed that although there was no difference between Instagram users and nonusers in terms of body dissatisfaction, Instagram users reported greater body monitoring than nonusers [6,7]. However, each of these works studied these conditions separately, and the combination of these conditions and how they are impacted by social media use has been overlooked. Given this, it becomes evident that there is a pressing need to conduct more in-depth investigations into the specific factors related to social media use that contribute to the development or exacerbation of these mental health concerns.

Family functioning pertains to how well family members fulfill their responsibilities and navigate life challenges [8]. It involves their interactions, growth, and responses to external influences [9]. Research highlights the need to investigate the potential risks posed by mobile device use to family functioning [10,11]. This suggests that families with flexibility and limited mobile device use may experience better cohesion and functioning, emphasizing the necessity for a more in-depth examination of the relationship between family functioning and social media use. Excessive social media use may lead to decreased emotional well-being, which negatively affects relationships [12]. For example, recent Indonesian research connects social media addiction to mild depression in university students, emphasizing the importance of bracing mental health by promoting family relationships and religiosity while addressing social media overuse [13]. Another study of mental health outpatients suggested a potential association between family functioning and psychological distress [14]. Although research has explored the link between social media and factors such as social connectedness, friendship quality, emotional well-being, and interpersonal relationships [15,16], there is a research gap regarding the impact of social media use on family functioning. Thus, it is imperative to conduct comprehensive research on family functioning and mental health, while analyzing their correlation with social media use.

In this paper, we looked at the relationships between social media use, mental health, and family functioning. Specifically, we examined various mental health conditions such as anxiety, self-esteem, depression, body dysmorphia, addiction to social media, and eating disorders. We selected Saudi Arabia as our sample for this study because of the increasing rates of internet penetration and social media use (90%) [17]. This high prevalence provides a valuable opportunity to examine the potential impact of social media on mental health and family functioning in this specific cultural context. Family is a fundamental unit in Saudi Arabian culture, and understanding the dynamics altered by social media is essential to comprehending broader societal changes. Investigating this aspect will not only enrich the existing literature, but also provide valuable insights into the evolving role of technology in shaping familial relationships and dynamics within the Saudi Arabian context. Thus, Saudi Arabia offers a unique setting characterized by a blend of traditional values, rapid modernization, and an evolving digital landscape. These factors create an intriguing environment in which to investigate the effects of social media on mental health and family dynamics. Two studies on social media use in Saudi Arabia’s web-based community have been conducted, one highlighting the mental health problems associated with social media use [3] and the influence of social media on food consumption among individuals [18]. To our knowledge, there is no research on how social media use may affect family functioning in Saudi Arabia. Thus, our study aimed to provide insights tailored to this cultural context. We aimed to investigate the relationship between social media use and mental health outcomes, with a particular focus on age and gender differences. Gender plays a significant role in this context, and research indicates that women are more likely than men to experience these mental health issues and they also tend to use social media platforms more frequently than their male counterparts [19,20].

## Methods

### Procedures

The data collected in this study were gathered through a subsection of an anonymous web-based survey titled “The Effect of COVID-19 on Social Media Usage, Mental Health, and Family Functioning.” The survey was designed based on a review of previous studies and surveys on pandemic effects [21-24]. Recruitment for this survey used diverse web-based social media platforms such as Facebook and Twitter. The survey was distributed to participants in Saudi Arabia with a clear emphasis on targeting a Saudi sample. We obtained informed consent from all participants before they completed the survey. The survey was designed to be anonymous and voluntary. Participants were given the option to answer the survey in English or Arabic. The survey targeted social media users who were at least 18 years old, and included sections on demographics, COVID-19, social media use, and well-being. Refer to Multimedia Appendix 1 for more information on the survey.

Participants were asked to choose which of the following prominent social networking sites they had an account on: Instagram, Facebook, Twitter, Snapchat, Google+, Vine, Tumblr, Pinterest, YouTube, and others. Participants were also provided with the option to specify “Other” platforms or “I do not have any social media accounts.” Participants were asked to estimate how much time they would spend on each platform during a typical day of browsing. No time (0), <5 minutes (1), 5-15 minutes (2), 15-30 minutes (3), 30 minutes -1 hour (4), 1-2 hours (5), 4-6 hours (7), 6-8 hours (8), 8-10 hours (9), or >10 hours (10). Participants were asked to respond to a variety of questions regarding the activities they engaged in on social media in general (not on specific platforms). Only participants who indicated that they had at least one social media account were presented with questions on social media activities. The web-based survey used in this study, designed based on a review of previous studies and surveys on the pandemic, can serve as a model for future research on the effects of social media use on mental health and family functioning.

### Measures

#### Mental Health Status

We used the General Health Questionnaire-12 (GHQ-12), a well-established 12-item self-report assessment tool, to assess the mental health status of the survey participants. The Likert Scale was used to score all 12 questions in the GHQ-12, generating 3 distinct statistical indicators: typical, suggestive evidence of distress, and severe problems with psychological distress. This tool is widely used to assess psychological distress and mental well-being. The validity and reliability of this scale have been demonstrated [25-27].

#### Family Functioning Status

We used the Family Assessment Device Questionnaire (FAD) to estimate participants’ family functioning status. FAD is a self-reported scale specifically designed to provide insights into the overall dynamics and functionality within a family. It assesses family relationships and identifies areas of potential dysfunction by adhering to the McMaster Model of Family Functioning. Within the scope of this study, participants were presented with the general functioning scale of the FAD, comprising 12 questions and yielding 4 distinct statistical outputs: healthy, almost healthy, almost unhealthy, and unhealthy. FAD was chosen for this study because it best suited the study objectives and demonstrated its validity and reliability [28-30].

#### Mental Health Conditions

We present 6 mental health conditions—anxiety, self-esteem, depression, body dysmorphia, addiction to social media, and eating disorders—collected through direct survey questions where participants self-reported whether social media affected those conditions. The inclusion of these conditions in our study is justified based on their prevalence, established links to social media use, public health importance, diverse impacts on mental health, and practical implications for interventions and policies [31-34]. Notably, these issues are dominant, with a high incidence of social media addiction among individuals experiencing these mental health challenges [35].

### Pilot Testing

To assess the initial survey, 4 participants participated in a pilot test. User feedback was collected to identify potential problems. The study had improved readability and validity because of this iterative process.

### Statistical Analysis

Descriptive statistics were reported as numbers and percentages for categorical variables. The mean and SD are reported for the numerical values. A score was calculated based on the frequency of social media access and the average time spent on social media. The participants were classified based on their scores into 4 quartiles (Q1-Q4). Participants in the first quartile had the lowest social media use, whereas those in the fourth quartile had the highest social media use. To calculate the mental health score for the GHQ-12, we summed the assigned values (0-3) for each response, with higher scores indicating a greater likelihood of mental health issues. For family functioning using the FAD, we assigned values (1-4) to each response, with higher scores indicating a greater likelihood of unhealthy family functioning. To assess self-reported mental health conditions, including anxiety, self-esteem, and depression, we analyzed respondent’s answers to identify correlations with their social media scores. A chi-square test was performed to determine the association between social media use, mental health, and family functioning scores. Logistic regression was performed to identify the factors associated with mental health and family functioning. SPSS 28 (IBM Corp) was used for the analysis, and statistical significance set at *P*<.05 is considered statistically significant.

### Recruitment

A total of 314 social media users who participated in this study were surveyed between the periods of 2021 to 2023 across 2 rounds to validate the results. Furthermore, 74.5% (n=234) were female, and 24.2% (n=76) were male. Most participants (n=293, 93.3%) were from Saudi Arabia, whereas the rest (n=21, 6.7%) represented other nationalities because of the nature of web-based sampling. The Saudi sample serves as an interesting case study for investigating the impact of social media on mental health and family functioning. First, Saudi Arabia is a highly conservative society that is undergoing rapid modernization, with social media playing a significant role in this transformation. According to recent statistics, 29.10 million social media users in Saudi Arabia access it through their mobile devices [17]. Second, there is a lack of research on the effects of social media on mental health and family functioning in Saudi Arabia. Finally, given that Saudi Arabia is a highly collectivist society, family dynamics play a significant role in shaping individual behaviors and attitudes, making it an ideal context to explore the interplay between social media use and family functioning. The largest group of participants was aged 35-44 years old (n=80, 25.5%), followed by 55-64 years (n=75, 23.9%). Regarding psychological and medical conditions, most respondents did not report having any psychological (n=271, 86.3%) or medical condition (n=216, 68.8%). Regarding the respondents’ educational background, the highest reported level of education was a bachelor’s degree (n=138, 43.9%), followed closely by a doctorate (n=81, 25.8%). A small proportion of respondents reported having a master’s degree (n=60, 19.1%), whereas a minority reported having a high school degree (n=26, 8.3%). Only a small percentage of the respondents reported having no formal education (n=9, 2.9%). Regarding participants’ race, most respondents identified as Arab (n=284, 90.4%), whereas a minority identified as non-Arab (n=30, 9.6%). Table S1 in Multimedia Appendix 2 provide more details on the sample demographics.

### Ethical Considerations

The Effect of COVID-19 on Social Media Usage, Mental Health, and Family Functioning survey was ethically approved by King’s College London ethics committee LRS-19/20-19717.

## Results

The purpose of this report was to summarize the results of a survey on the perceived impact of social media platforms on mental health.

### Social Media Use

The data show that most respondents accessed social media frequently, with (n=115, 36.6%) reporting that they go on social media every couple of hours. When asked about the amount of time they spent on social media per day, the most common response was 3-5 hours (n=86, 27.4%), followed by 1-2 hours (n=58, 18.5%). When asked about the duration of their social media sessions, most respondents reported spending approximately 15 minutes or less logged in (n=129, 41.1%). A significant majority of respondents (n=238, 75.5%) reported that they did not feel it was healthy to spend much time on the internet. Most respondents (n=267, 85.03%) reported accessing social media in the evening, whereas 25.48% (n=80) reported accessing it at midnight. When asked about their addiction to social media, 41.1% (n=129) of the respondents reported feeling addicted. Table 1 provides more details on the social media use of the participants. The most common reason for using social media was to keep in touch with friends and family (n=243, 77.39%). Other reasons included inspiration (n=160, 50.96%), browsing or wasting time (n=135, 42.99%), and entertainment (n=130, 41.4%). Around 29.94% (n=94) of the participants reported using social media for work or business purposes, and only 5.1% (n=16) reported using social media for dating or romantic purposes. Table 2 elaborates the reasons for using social media.

**Table 1:** Social media use among participants.

		Participants, n (%)
**How often currently do you go on social media?**		
	Almost never or rarely	1 (0.3)
	Just about every month	3 (1.0)
	Every couple of days	10 (3.2)
	Just about every day	83 (26.4)
	Every couple of hours	115 (36.6)
	Just about every hour	55 (17.5)
	Every couple of minutes	47 (15.0)
**On average how much time do you think you spend on social media per day? (h)**		
	<1	25 (8.0)
	1-2	58 (18.5)
	2-3	63 (20.1)
	3-5	86 (27.4)
	5-7	47 (15.0)
	>7	35 (11.1)
**Every time you log in to social media, on average how long do you spend logged in?**		
	About 15 min or less	129 (41.1)
	About 30 min	83 (26.4)
	About an hour	65 (20.7)
	More than an hour	37 (11.8)
**Do you feel it is healthy to spend that much time online?**		
	No	237 (75.5)
	Yes	77 (24.5)
**When do you currently access social media? (multiple answers allowed)**		
	Morning (from 5 AM to 11:59 AM)	149 (47.45)
	Afternoon (from 12 PM to 6 PM)	125 (39.81)
	Evening (from 6 PM to 11:59 PM)	267 (85.03)
	Midnight (exactly 12 AM to 4:59 AM)	80 (25.48)
**Do you consider yourself addicted to social media?**		
	No	185 (58.9)
	Yes	129 (41.1)

**Table 2:** Reasons for using social media.

What do you use social media for? (multiple answers allowed)	Participants, n (%)
Keeping in touch with friends and family	243 (77.39)
Event planning	49 (15.61)
Buying and selling	50 (15.92)
Inspiration	160 (50.96)
News about COVID-19 (coronavirus) pandemic	125 (39.81)
To make new friends	8 (2.55)
To find employment	13 (4.14)
To browse or time waste	135 (42.99)
To raise awareness	62 (19.75)
To provide support to others	58 (18.47)
To share your posts	66 (21.02)
To work	76 (24.20)
None of the above	3 (0.96)

### Participants Attitudes

When asked if social media distracted them when they needed to be productive, 33.8% (n=106) of the respondents reported that it did. In contrast, 66.2% (n=208) of the respondents reported that social media does not distract them when they need to be productive. The data also revealed that a significant majority of respondents (n=238, 75.5%) did not care about how many people like or view their posts or pictures, whereas 24.5% (n=77) of respondents reported that they do care. When asked about cyberbullying on social media, 12.1% (n=38) of the respondents reported that they had been cyberbullied in some way, whereas 87.9% (n=276) reported that they had not. In terms of how social media affects self-esteem, only 30.9% (n=97) of the respondents reported thinking negatively about their body when seeing pictures of a person who has the body type they desire. Similarly, only 14.3% (n=45) of respondents reported feeling depressed when seeing posts about intriguing events in other people’s lives. When asked if they accept friend requests or followers from people they do not know to appear more popular, 84.7% (n=266) of the respondents reported that they did not, whereas 15.3% (n=48) reported that they did.

### Social Media Impact via Self-Reporting

Participants were asked to rate each platform on a scale of 0 to 5, with 0 indicating that they did not use the platform and 5 indicating that it was the most positive social media platform in their opinion. After those who reported not using the platform, an average score was calculated based on each participant’s opinion, with a lower score indicating a negative impact and a higher score indicating a positive impact.

**Figure 1:**
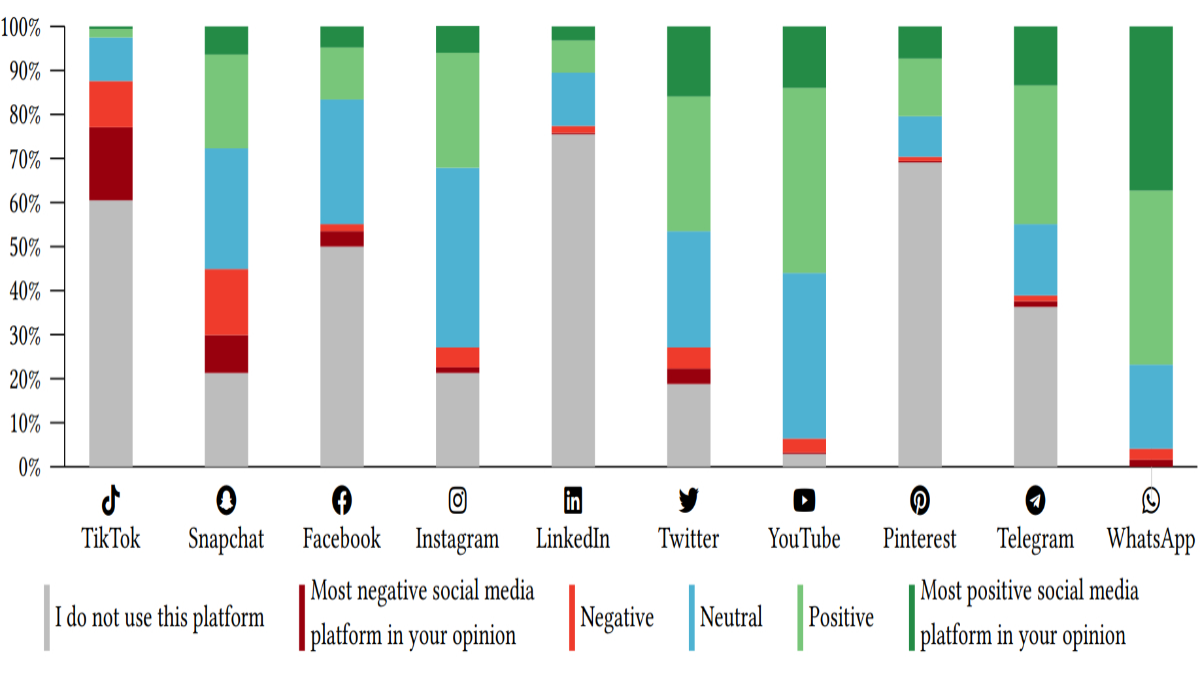
The perceived effect of each social media platform as indicated by the participants.

**Figure 2:**
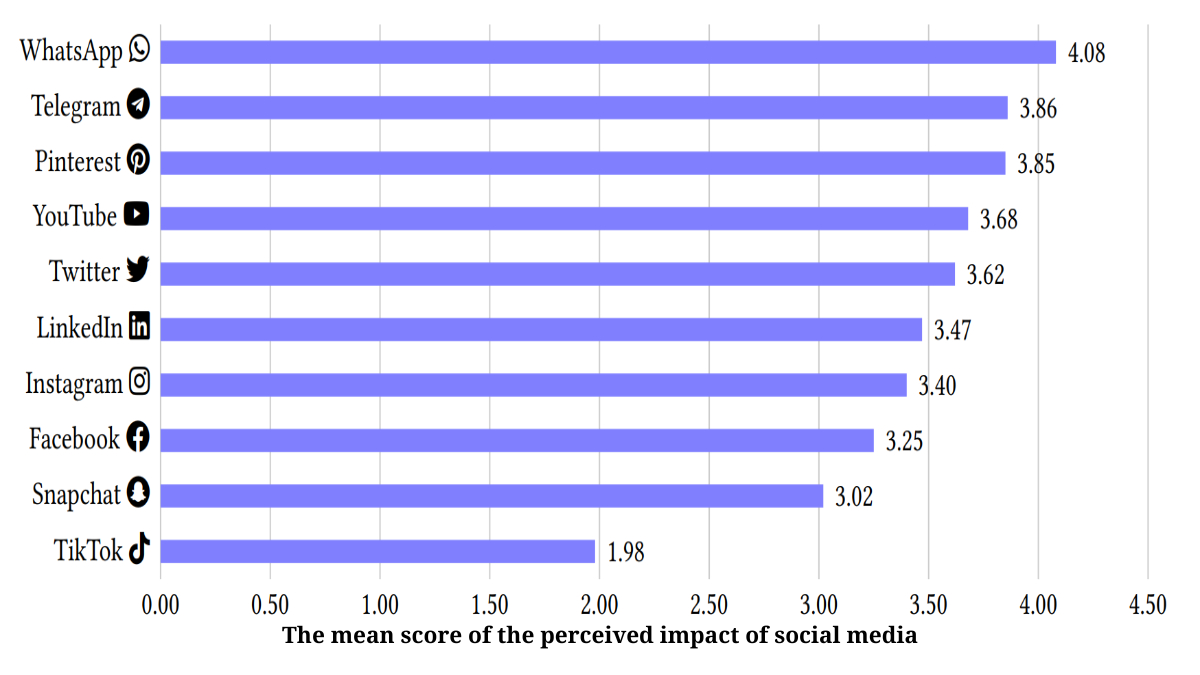
Mean scores for the perceived effect of social media platforms.

The platform perceived as having the most positive effect was WhatsApp (4.08), followed by Telegram (3.86), and Pinterest (3.85). The lowest score, indicating the most negative outcome, was observed for TikTok (1.98), followed by Snapchat (3.02). Table S2 in Multimedia Appendix 2 presents a comprehensive analysis of the perceived impact of each social media platform, as reported by the participants. The effects of individual platforms are shown in Figure 1. In addition, Figure 2 provides an overview of the mean scores indicating the perceived impact of social media platforms. Regarding self-reported mental health conditions, the most common effects were anxiety (n=217, 69.09%) and social media addiction (n=206, 65.45%). Other reported effects included depression (n=108, 34.55%), self-esteem (n=97, 30.91%), body dysmorphia (n=69, 21.82%), and eating disorders (n=46, 14.55%) as illustrated in Table 3. However, 25.45% (n=80) of the respondents who reported an impact stated that social media did not affect them. Finally, when asked about the emotions they experienced when using social networking sites, the most common responses were inspiration (n=159, 50.64%), motivation (n=83, 26.43%), and happiness (n=78, 24.84%). Other reported emotions included the fear of missing out (n=31, 9.87%), boosted self-esteem (n=64, 20.38%), jealousy (n=11, 3.5%), and rejection (n=9, 2.87%). Only 2.55% (n=8) of the respondents reported experiencing lower self-esteem when using networking sites. Table S3 in Multimedia Appendix 2 elaborates on social media use habits and their impact on participants.

Regarding the impact of social media on relationships with family members, 76.8% (n=241) of respondents reported that social media did not affect their relationships, whereas 23.2% (n=73) of respondents reported that it did. Among those who reported an impact, 15.3% (n=48) reported a positive effect, whereas 15.9% (n=50) reported a negative effect. When asked if they had a web-based persona, 91.1% (n=286) of the respondents reported that they did not, whereas only 8.9% (n=28) reported that they did.

**Table 3:** Social media impact.

What impact has social media had on your mental health?	Participants, n (%)
Anxiety	38 (69.09)
Self-Esteem	17 (30.91)
Depression	19 (34.55)
Body dysmorphia	12 (21.82)
Addiction to social media	36 (65.45)
Eating disorder	8 (14.55)
It has not affected me	14 (25.45)
None of the above	22 (40.00)

### Social Media Impact via Statistical Tools

Regarding mental health, most respondents (n=274, 87.3%) had a score that indicated mental health within the normal range, whereas (n=40, 12.7%) had a score that indicated affected mental health. For family functioning, most respondents (n=269, 85.7%) had a score that indicated healthy or almost healthy family functioning, whereas (n=45, 14.3%) had a score that indicated unhealthy or almost unhealthy family functioning. Table 4 shows the mental health and family functioning of the participants.

Statistical analysis using the chi-square test showed a statistically significant association between social media use and mental health (*P*<.001). Participants in the higher social media quartiles had a higher percentage of affected mental health (26.7% for the fourth quartile and 14.6% for the third quartile) as compared with participants in lower quartiles of social media use (9.1% in the first quartile and 4.8% in the second quartile). Table 5 shows the association between social media use and mental health. Statistical analysis using the chi-square test showed a statistically significant association between social media use and family functioning (*P*<.001). Participants in the higher social media quartiles had a higher percentage of unhealthy or almost unhealthy family functioning (30% for the fourth quartile and 14.6% for the third quartile) as compared with participants in the lower quartiles of social media use (9.1% for the first quartile and 8.3% for the second quartile). Table 6 illustrates the association between social media use and family functioning. Logistic regression was performed to identify the factors associated with mental health. Age and sex showed statistically significant results in the multivariate analysis. Female participants were more likely to have affected mental health as compared with male participants (odds ratio [OR] 4.69, 95% CI 1.42-15.49; *P*=.01).

For age participants who were between 25 and 34 years were more likely to have affected mental health as compared with participants who were 18 to 24 years (OR 6.10, 95% CI 1.42-26.15; *P*=.02). Table 7 illustrates the factors associated with affected mental health.

Logistic regression was applied to identify the factors associated with unhealthy or unhealthy family functioning. The age and social media use quartiles showed statistically significant differences. For gender, female participants were more likely to have unhealthy or almost unhealthy family functioning as compared with male participants (OR 3.32, 95% CI 1.17-9.46; *P*=.02). Regarding social media use quartiles, participants in the fourth quartile were more likely to have unhealthy or almost unhealthy family functioning as compared with participants in the first quartile (OR 4.22, 95% CI 1.45-12.31; *P*=.008). Table 8 illustrates the factors associated with unhealthy or almost unhealthy family functioning.

**Table 4:** Mental health and family function of participants.

			Participants, n (%)
**Mental health**			
	Affected	40 (12.7)	
	Within normal range	274 (87.3)	
**Family functioning**			
	Healthy or almost healthy	269 (85.7)	
	Unhealthy or almost unhealthy	45 (14.3)	

**Table 5:** Association between social media use and mental health.

Social media use quartiles	Mental health			*P* value
	Within normal range, n (%)	Affected, n (%)		
Q1	80 (90.9)	8 (9.1)	<.001	
Q2	80 (95.2)	4 (4.8)	<.001	
Q3	70 (85.4)	12 (14.6)	<.001	
Q4	44 (73.3)	16 (26.7)	<.001	

**Table 6:** Association between social media use and family functioning.

Social media use quartiles	Family functioning		*P* value
	Healthy or almost healthy, n (%)	Unhealthy or almost unhealthy, n (%)	
Q1	80 (90.9)	8 (9.1)	<.001
Q2	77 (91.7)	7 (8.3)	<.001
Q3	70 (85.4)	12 (14.6)	<.001
Q4	42 (70)	18 (30.0)	<.001

**Table 7:** Factors associated with affected mental health.

			Univariate				Multivariable		
			Odds ratio (95% CI)		*P* value	Odds ratio (95% CI)			*P* value
**Age (y)**									
	18-24	1 (N/A^a^)		N/A		1.00 (N/A)		N/A	
	25-34	4.77 (1.29-17.68)		.02		6.10 (1.42-26.15)		.02	
	35-44	1.65 (0.43-6.32)		.47		1.74 (0.42-7.27)		.45	
	45-54	0.97 (0.22-4.36)		.97		1.31 (0.26-6.66)		.75	
	55-64	0.43 (0.08-2.25)		.32		0.39 (0.06-2.44)		.32	
	65-74	0.00 (0.00)		<.99		0.00 (0.00)		<.99	
**Gender**									
	Male	1.00 (N/A)		N/A		1.00 (N/A)		N/A	
	Female	3.27 (1.13-9.52)		.03		4.69 (1.42-15.49)		.01	
	Prefer not to say	0.00 (0.00)		<.99		0.00 (0.00)		<.99	
**Residency**									
	In Saudi Arabia	1.00 (N/A)		N/A		1.00 (N/A)		N/A	
	Outside Saudi Arabia	0.71 (0.16-3.15)		.65		0.96 (0.16-5.68)		.96	
**Level of education**									
	High School	1.00 (N/A)		N/A		1.00 (N/A)		N/A	
	Bachelor’s degree	0.67 (0.23-1.99)		.47		0.34 (0.10-1.23)		.10	
	Master’s degree	0.55 (0.16-1.94)		.36		0.21 (0.04-1.00)		.05	
	Doctorate degree	0.34 (0.09-1.21)		.09		0.25 (0.06-1.15)		.08	
	None of the above	2.10 (0.39-11.43)		.39		1.86 (0.21-16.73)		.58	
**Social media use quartiles**									
	Q1	1.00 (N/A)		N/A		1.00 (N/A)		N/A	
	Q2	0.28 (0.11-0.69)		.006		0.41 (0.11-1.60)		.20	
	Q3	0.14 (0.04-0.44)		.001		1.47 (0.49-4.47)		.49	
	Q4	0.47 (0.20-1.09)		.08		2.36 (0.78-7.16)		.13	
**Do you consider yourself addicted to social media?**									
	No	1.00 (N/A)		N/A		1.00 (N/A)		N/A	
	Yes	0.59 (0.30-1.15)		.12		0.83 (0.36-1.94)		.67	

^a^N/A: not applicable.

**Table 8:** Factors associated with unhealthy or almost unhealthy family functioning.

			Univariate					Multivariable		
			Odds ratio (95% CI)		*P* value		Odds ratio (95% CI)			*P* value
**Age (y)**										
	18-24	1 (N/A^a^)		N/A		1.00 (N/A)			N/A	
	25-34	1.24 (0.42-3.69)		.69		1.43 (0.40-5.09)			.58	
	35-44	0.82 (0.28-2.41)		.72		0.83 (0.25-2.77)			.77	
	45-54	0.86 (0.28-2.66)		.79		1.08 (0.29-4.07)			.90	
	55-64	0.41 (0.12-1.37)		.15		0.39 (0.09-1.62)			.19	
	65-74	0.00 (0.000)		<.99		0.00 (0.00)			<.99	
**Gender**										
	Male	1.00 (N/A)		N/A		1.00 (N/A)			N/A	
	Female	2.84 (1.08-7.49)		.04		3.32 (1.17-9.46)			.02	
	Prefer not to say	4.73 (0.41-54.20)		.21		3.89 (0.29-52.68)			.31	
**Residency**										
	In Saudi Arabia	1.00 (N/A)		N/A		1.00 (N/A)			N/A	
	Outside Saudi Arabia	0.28 (0.04-2.16)		.22		0.46 (0.05-4.04)			.48	
**Level of education**										
	High School	1.00 (N/A)		N/A		1.00 (N/A)			N/A	
	Bachelor’s degree	0.75 (0.26-2.22)		.61		0.51 (0.15-1.68)			.27	
	Master’s degree	0.30 (0.07-1.23)		.09		0.27 (0.06-1.26)			.10	
	Doctorate degree	0.73 (0.23-2.31)		.59		0.89 (0.24-3.27)			.86	
	None of the above	2.10 (0.39-11.43)		.39		3.36 (0.45-24.99)			.24	
**Social media use quartiles**										
	Q1	1.00 (N/A)		N/A		1.00 (N/A)			N/A	
	Q2	0.91 (0.31-2.63)		.86		0.94 (0.30-2.93)			.92	
	Q3	1.71 (0.66-4.43)		.27		1.89 (0.65-5.46)			.24	
	Q4	4.29 (1.72-10.68)		.002		4.22 (1.45-12.31)			.008	
**Do you consider yourself addicted to social media?**										
	No	1.00 (N/A)		N/A		1.00 (N/A)			N/A	
	Yes	1.45 (0.77-2.73)		.25		0.72 (0.33-1.58)			.42	

^a^N/A: not applicable.

## Discussion

### Principal Findings

This study’s insights, grounded in a sample from Saudi Arabia, provide a culturally specific lens at the intersection of social media use and mental health. The prevalence of frequent social media access and reported durations align with the global trend [36], highlighting the pervasive nature of these platforms in the Saudi context. However, it is crucial to interpret these findings within the cultural framework of Saudi Arabia, where familial and social ties are of significant importance [37]. The primary motivation for social media use, namely staying connected with friends and family, resonates strongly with the cultural emphasis on community bonds in Saudi society. This underscores the integral role that social media plays in facilitating and maintaining relationships, which is a culturally significant function. The findings of our study suggest that social media is a popular and frequently used technology in Saudi Arabia, with a significant proportion of users expressing concern about their use habits. Unlike previous research, which analyzed social media platforms and their effect on mental health [38-41], our study applied an in-depth investigation across platforms to evaluate each platform’s impact on mental health. We first tested the effect of time spent on each platform by participants and found no particular association between time spent on various platforms and mental health. Despite small negative correlations between time spent on YouTube, Instagram, and Snapchat and body satisfaction and a small positive correlation between time spent on YouTube and depressive symptoms. Our findings imply that future research might benefit from changing attention from time spent generally perusing platforms to participant’s attitudes when engaging with social media platforms. The platform-specific analysis, with WhatsApp emerging as the most positively perceived and TikTok as the most negatively perceived, was influenced by cultural preferences and content norms in the Saudi context. Understanding these variations is essential for tailoring interventions and guidelines to align with the cultural values and sensitivities of the Saudi population.

Participants’ attitudes toward social media platforms may have affected their mental health. For instance, these platforms may be a hotspot for frequent and unjustified comparisons of appearances, which might be harmful to mental health. Performing more appearance comparisons with others and thinking that others are more attractive than you are on social media were both independent predictors of lower body satisfaction, more eating disorders, and higher levels of eating disorders. Our results support previous research on teenagers and adults, emphasizing the significance of appearance comparisons as a potential mechanism through which social media use may be detrimental to mental health [42,43]. Our results further imply that, despite their emphasis on physical appearance, these comparisons may have a detrimental effect on issues that are not just related to beauty, such as body satisfaction and eating disorders, but also on general mental health (such as depressive symptoms and anxiety). In our results, compared with men, women frequently paid greater attention to and regarded their beauty as a measure of their self-worth. Therefore, women may be less satisfied with their appearance and more depressed than men are. This is because women may engage in more frequent appearance comparisons on social media [44,45]. The identified effects on mental health, particularly anxiety and addiction, have cultural implications. Given the societal importance placed on mental well-being in Saudi Arabia, these findings underscore the need for targeted mental health awareness and support initiatives within a cultural context.

Logistic regression findings indicate that age and gender are factors associated with affected mental health and unhealthy family functioning. This is in line with a study that emphasized the significance of demographic factors when studying mental health in a Saudi sample [46]. According to our logistic regression findings, age and gender significantly influenced mental health and family functioning in the Saudi context. Female participants had a higher likelihood of experiencing mental health issues (OR 4.69, 95% CI 1.42-15.49; *P*=.01), emphasizing the need for gender-specific support. In addition, participants aged 25-34 years were more likely to face mental health challenges than those aged 18-24 years (OR 6.10, 95% CI 1.42-26.15; *P*=.02), suggesting the importance of age-targeted interventions. In terms of family functioning, female participants were more likely to report unhealthy dynamics (OR 3.32, 95% CI 1.17-9.46; *P*=.02), whereas older individuals in higher social media use quartiles were more likely to experience such challenges (OR 4.22, 95% CI 1.45-12.31; *P*=.008). Recognizing these age and gender dynamics is vital for tailoring mental health and family support strategies in Saudi Arabia. The statistical tools revealed associations between social media use and mental health, as well as family functioning, emphasizing the need for culturally informed strategies to address potential challenges. The statistical analysis revealed significant associations between social media use, mental health, and family functioning within the Saudi Arabian context, underscoring the importance of culturally informed strategies. Higher social media quartiles exhibited a notable correlation with a greater likelihood of affecting mental health and unhealthy family functioning. These findings emphasize the nuanced interplay between web-based activities and individual well-being as well as the broader impact on familial relationships. Considering these associations, it is crucial to develop interventions and support mechanisms that are culturally sensitive and tailored to the unique sociocultural dynamics of Saudi Arabia. Recognizing the intricate relationship between social media use and mental health outcomes, along with its implications for family functioning, is the key to fostering digital well-being in this cultural context.

### Limitation and Implications

It is important to consider the following limitations when interpreting the results of our study. More in-depth longitudinal studies are needed to explore the association between social media use and mental health over time. The sample’s specificity of the sample to Saudi Arabia’s demographic and cultural context may restrict the generalizability of the results to more diverse populations. To enhance the external validity, future research should aim for a broader and more representative sample that encompasses a range of cultural, socioeconomic, and demographic backgrounds. The study’s implications of this study are multifaceted and have significant relevance for the development of targeted interventions and public health initiatives in Saudi Arabia. First, the identified associations between social media use and mental health outcomes underscore the need for specific culturally sensitive interventions. Tailored mental health programs can address the distinct challenges faced by different demographic groups, such as female participants and individuals aged 25 to 34 years, who were found to be more susceptible to affected mental health. These interventions could include educational campaigns, support groups, and digital resources tailored to the cultural nuances of the Saudi context. Moreover, the observed link between social media use and family functioning emphasizes the interconnected nature of web-based behavior and familial relationships. Culturally informed strategies should not only address individual well-being, but also promote healthier family dynamics in the digital age. Public health campaigns can play a pivotal role in raising awareness of the potential impact of social media on family relationships and fostering open discussions within families and communities about responsible digital practices. This study not only contributes to the global discourse on social media, mental health, and family functioning but also offers nuanced insights specific to Saudi Arabia. Recognizing and understanding these cultural nuances are paramount for developing effective policies, educational programs, and support systems that promote positive mental health outcomes tailored to the sociocultural landscape of Saudi Arabia.

### Conclusions

This study investigated the perceived impact of social media platforms on mental health and family functioning in a Saudi Arabian sample. The findings reveal important insights with implications for public health initiatives and targeted interventions. This study highlighted the observable association between social media use, mental health, and family functioning. Notably, age and gender have emerged as significant factors influencing mental health and unhealthy family functioning. This underscores the necessity for culturally sensitive strategies to address these identified challenges and tailor interventions to the specific needs of different demographic groups. Recognizing the nuanced associations observed in this study can inform the development of interventions that promote digital well-being, considering the crucial role of familial ties in the societal framework of Saudi Arabia.

## Appendix

Multimedia Appendix 1 Effect of COVID-19 on social media use, mental health, and family functioning survey.

Multimedia Appendix 2 Statistical analysis tables.

## Abbreviations

FAD: Family Assessment Device Questionnaire
GHQ-12: General Health Questionnaire-12
OR: odds ratio

## Data Availability

The data sets generated during and analyzed during this study are available from the author upon reasonable request.
