# Supplementary material for: The Impact of Social Media Use on Mental Health and Family Functioning Within Web-Based Communities in Saudi Arabia: Ethnographic Correlational Study
Source: JMIR Form Res. 2024 Jan 16;8:e44923. doi: 10.2196/44923 (PMC10828947; doi:10.2196/44923)
Supplement: Multimedia Appendix 1 [file formative_v8i1e44923_app1.docx]

The effect of COVID-19 on social media usage, mental health, and family

functioning

INFORMATION SHEET FOR PARTICIPANTS

Ethical Clearance Reference Number: LRS-19/20-19717 Version Number: 27/July/2020

Title of study:

The effect of COVID-19 on social media usage, mental health, and family functioning.

Invitation paragraph:

I would like to invite you to participate in this research project which forms part of my PhD thesis research. Before you decide whether you want to take part, it is important for you to understand why the research is being done and what your participation will involve. Please take the time to read the following information carefully and discuss it with others if you wish. Ask me if there is anything that is not clear or if you would like more information.

What is the purpose of the study?

The purpose of the study is understanding to what extent COVID-19 (Coronavirus) pandemic affects a person's social media platforms use, mental health, and family functioning. In addition to exploring to what extent cultural differences influence this effect. By collecting real-time data, the researcher hopes that the results will inform future researchers in the field, social media users, mental health society, and the community as a whole understanding the effect of COVID-19 (Coronavirus) pandemic on social media platforms use, mental health, and family functioning.

Why have I been invited to take part?

You are being invited to participate in this study for us to collect real-time data to help us understand how COVID-19 (Coronavirus) pandemic is affecting social media usage, mental health, and family functioning. We are looking for volunteers aged 18 years or older.

What will happen if I take part?

If you agree to take part, you will complete a survey. The survey will ask you demographic, social media, mental health, and family functioning questions. The survey will take you approximately about 20 minutes to complete. You will be presented with the option of providing your email address for the researcher to resend the survey after the passing of 30 days from the first round of data collection. The purpose behind that is to detect changes as time passes in the results of this study if there's is any or confirm its findings from the first round of data collection. You can refuse to provide your email address. All email addresses, if offered, will be separated automatically from the research data, will be deleted after the last round of data collection, and will not be used for any other purpose but to resend the survey. This ensures that your answers to the survey questions are truly

anonymous.

Do I have to take part?

Participation is completely voluntary. You should only take part if you want to and choosing not to take part will not disadvantage you in any way. If you choose to take part you will be asked to provide your consent. To do this you will be asked to indicate that you are above 18 years old and you have read and understood the information provided and that you consent to your data being used for the purposes explained.

You are free to withdraw at any point during the completion of the survey, without having to give a reason by simply not submitting the survey. Withdrawing from the study will not affect you in any way. Once you submit the survey, it will no longer be possible to withdraw from the study because the data will be anonymous. Please do not include any personally identifiable information in your responses.

What are the possible risks of taking part?

Although we do not anticipate any risks to your mental health or wellbeing from participating in the study links to online wellbeing support will be provided in the survey to all participants.

Data handling and confidentiality:

This research is anonymous. This means that nobody, including the researchers, will be aware of your identity, and that nobody will be able to connect you to the answers you provide, even indirectly. Your answers will nevertheless be treated confidentially and the information you provide will not allow you to be identified in any research outputs/publications. The data controller for this project will be King’s College London

(KCL). Research is a task that the University carries out in the public interest. Your data will be processed in accordance with the standards set by the General Data Protection Regulation 2016 (GDPR) please visit the link below: [https://www.kcl.ac.uk/research/support/research-ethics/kings-college-london-statement-](https://www.kcl.ac.uk/research/support/research-ethics/kings-college-london-statement-on-use-of-personal-data-in-research) [on-use-of-personal-data-in-research](https://www.kcl.ac.uk/research/support/research-ethics/kings-college-london-statement-on-use-of-personal-data-in-research)

What if I change my mind about taking part?

You are free to withdraw at any point in the project, without having to give a reason. Withdrawing from the project will not affect you in any way. You are able to withdraw your data from the project by not completing and not submitting the survey answers, after which withdrawal of your data will no longer be possible because users can not be identified from the data set. If you do not complete and submit the survey, your responses will be automatically deleted. Backtrack throughout the survey is available if you needed to change your answers.

What will happen to the results of the study?

The results of the study will be used for academic research. In order to understand the impact COVID-19 has on your social media usage, mental health, and family functioning. The data you provide may also be used for other research purposes in the future. The researcher aims to publish the results of this research in scientific journals, conferences, and public outreach events.

Who should I contact for further information?

If you have any questions or require more information about this study, please contact me using the following contact details:

The Centre for Urban Science and Progress laboratory (CUSP) Department of Informatics

King’s College London Bush House – 30 Aldwych London WC2B 4BG

United Kingdom

Email: [bdour.alwuqaysi@kcl.ac.uk](mailto:bdour.alwuqaysi@kcl.ac.uk)

What if I have further questions, or if something goes wrong?

If this study has harmed you in any way or if you wish to make a complaint about the conduct of the study you can contact King's College London using the details below for further advice and information:

Supervisor: Dr.Rita Borgo Department of Informatics King’s College London Bush House – 30 Aldwych London WC2B 4BG

United Kingdom

Email: [rita.borgo@kcl.ac.uk](mailto:rita.borgo@kcl.ac.uk)

Thank you for reading this information sheet and for considering taking part in this research.

* Indicates required question

# Do you consent for the researcher to recontact you by email for the purposes *

outlined in the information sheet?

## Mark only one oval.

Yes, I do. No, I do not.

# By ticking the below box I conﬁrm that I am 18 years old or above, I have read *

and understood the information provided to me, and I agree to take part in this research project.

*Check all that apply.*

I am 18 years old or above, and I consent to participate in this study.

First Demographic Questions

3. 1- Age: *

## Mark only one oval.

18-24 years old

25-34 years old

35-44 years old

45-54 years old

55-64 years old

65-74 years old 75 years or older

# 2- Gender: *

## Mark only one oval.

Female Male

Prefer not to say

# 3- The country you are currently living in: *

## Mark only one oval.

United Kingdom Saudi Arabia Afghanistan Albania

Algeria Australia Bangladesh Belgium Brazil Bulgaria Canada China Colombia Croatia Cuba Denmark Egypt Ethiopia France Germany Greece India Indonesia Iran

Iraq Ireland Italy Jamaica Japan Jordan Kenya Kuwait

Lebanon Libya Malaysia Mexico Morocco Netherlands New Zealand Nigeria Norway Pakistan Panama Paraguay Philippines Portugal Romania Russia Scotland Senegal Serbia Singapore Slovakia South Africa South Korea Spain

Sri Lanka Sudan Sweden Switzerland Syria Taiwan Tajikistan Thailand Tonga Tunisia

Turkey Ukraine

United Arab Emirates United States Uruguay

Venezuela Vietnam Wales Zambia Zimbabwe

None of the above

# 4- Do you suffer from psychological condition? *

## Mark only one oval.

Yes No

Not sure

# 5- Do you suffer from medical condition? *

## Mark only one oval.

Yes No

Not sure

# 6- What would best describe you? *

## Mark only one oval.

White (English, Welsh, Scottish, Northern Irish, British, Irish, Gypsy or Irish Traveller, Any other White background)

Asian (Indian, Pakistani, Bangladeshi, Chinese, any other Asian background) Black (African, Caribbean, any other Black background)

Arab

Mixed / Multiple ethnic groups (White and Black Caribbean, White and Black African, White and Asian, any other Mixed Multiple ethnic backgrounds)

Any other ethnic group

# 7- What type of place do you live in? *

## Mark only one oval.

Villa or mansion House in a compound

House with garden or backyard House without garden or backyard Flat in a flat block

Student halls

Room(s) in a shared house (for example as a lodger) None of the above

# 8- Which of these ﬁelds’ best describes your major or your ﬁeld of work? *

## Mark only one oval.

Agriculture, Agriculture Operations, And Related Sciences Arabic Language

Biological And Biomedical Sciences

Business, Management, Marketing, And Related Support Services Communication, Journalism, And Related Programs Communications Technologies/Technicians And Support Services Computer And Information Sciences And Support Services Education

Engineering Technologies And Engineering-Related Fields English Language And Literature/Letters

Family And Consumer Sciences/Human Sciences Foreign Languages, Literatures, And Linguistics Health Professions And Related Programs History

Homeland Security, Law Enforcement, Firefighting, and Related Protective Services

Legal Professions And Studies

Liberal Arts And Sciences, General Studies And Humanities Library Science

Mathematics And Statistics

Mechanic And Repair Technologies/Technicians Military Technologies And Applied Sciences Natural Resources And Conservation Philosophy And Religious Studies

Physical Sciences Psychology

Public Administration And Social Service Professions Science Technologies/Technicians

Social Sciences

Visual And Performing Arts None of the above

# 9- Level of Education (you can indicate the level of education during the period *

you are in):

## Mark only one oval.

High School Bachelor’s degree Master’s degree Doctorate degree None of the above

Second COVID-19 (Coronavirus) pandemic Questions

1. 1- How would you rate your knowledge level on COVID-19? *

*Mark only one oval.*

1 2 3 4 5

Very Very good knowledge

poo

r kno

wled

ge


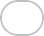

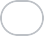

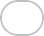

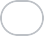

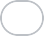


# 2- Are you following the recommendations from authorities to prevent the *

spread of COVID-19?

*Mark only one oval.*

1 2 3 4 5

Non Very much so

e at

all


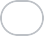

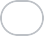

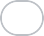

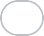

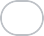


# 3- How much conﬁdence do you have in the government within the country you *

are currently in that can handle COVID-19 well?

*Mark only one oval.*

1 2 3 4 5

e at

all


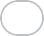

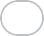

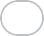

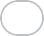

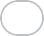


Non

A lot

# 4- How much conﬁdence do you have that the health service within the country *

you are currently in that can cope during COVID-19?

*Mark only one oval.*

1 2 3 4 5

e at

all


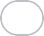

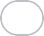

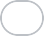

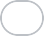

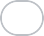


Non

A lot

# 5- How much conﬁdence do you have that essentials (for example access to *

food, water, medicines, and deliveries) will be maintained during COVID-19?

*Mark only one oval.*

1 2 3 4 5

e at

all


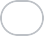

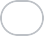

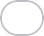

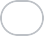

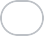


Non

A lot

# 6- Have you had COVID-19 (coronavirus)? *

## Mark only one oval.

Yes diagnosed and recovered Yes diagnosed and still ill

Not formally diagnosed but suspected No, I have not

# 7- What does the COVID-19 (coronavirus) pandemic mean to you? Please *

describe in 1-3 words

# 8- Have you chosen to live somewhere different from your usual *

accommodation during the COVID-19 (coronavirus) pandemic?

## Mark only one oval.

Yes No

# 8.1- If yes, why have you chosen to live somewhere different from your usual accommodation? (Choose as many as applicable)

*Check all that apply.*

To protect my usual household To care for someone

For practical reasons (for example to be closer to work) For financial reasons

For comfort or company (for example more space, to be with a friend, partner, or family)

For safety (for example did not feel safe in usual accommodation) So that someone could care for me

It was not my decision None of the above reasons

# 9- How often have you been anxious about COVID-19? *

## Mark only one oval.

Not at all

For several days since the beginning of the pandemic

For more than half the days since the beginning of the pandemic Nearly every day since the beginning of the pandemic

# 10- Have any of the following been worrying you at all, even if only in a minor *

way during COVID-19 pandemic? (Choose as many as applicable)

*Check all that apply.*

Marriage or other romantic relationship Friends or family living in your household Friends or family living outside your household Neighbors

Work (even if you feel your job is safe) Losing your job/unemployment Finances

Getting medication Getting food

Your own safety/security Internet access Boredom

The change of routine and the way we live our life Future plans

Infected with COVID-19

Becoming seriously ill from COVID-19 Worrying about your pet

None of these

# 11- Before the pandemic, how often do you meet up with people face to face *

for work?

## Mark only one oval.

Every day

Three or more times a week Once or twice a week

Once or twice a month Less than once a month

# 12- Before the pandemic, how often do you meet up with people face to face *

socially, not for work (for example friends, family, relatives, or social events with colleagues)?

## Mark only one oval.

Every day

Three or more times a week Once or twice a week

Once or twice a month Less than once a month

# 13- What is your current isolation status? *

## Mark only one oval.

I am worried about getting infected with COVID-19 and I have an existing medical condition or I am categorized as a high risk

I am worried about getting infected with COVID-19 but I am not a high risk I wish to protect/shield a family member/friend who is a high risk

I am worried about spreading COVID-19 to others

It has been ordered by the government (for example as part of lockdown) Another reason not relating to COVID-19 (for example maternity leave, pre-

existing illness, or disability) None of the above

# 14- Have you experienced any of the following due to or during the COVID-19 *

pandemic? (Choose as many as applicable)

*Check all that apply.*

Lost your job/been unable to do paid work

Your spouse/partner lost their job or was unable to do paid work

Major cut in household income (for example due to you or your partner being furloughed/put on leave/not receiving sufficient work)

Divorce and family dispute Unable to pay bills/rent/mortgage Evicted/lost accommodation Unable to access sufficient food

Unable to access the required medication

Canceled operations or difficulty making doctor appointments

Somebody close to you is ill in hospital (due to COVID-19 or another illness) You lost somebody close to you (due to COVID-19 or another cause)

None of the above

# 15- Has anything helped you cope day-to-day during the COVID-19 pandemic? *

(for example learning to manage anxiety better, support from the community, etc.). Would you like to tell us more about this?

# 16- How has your sleep been during the COVID-19 pandemic? *

## Mark only one oval.

Very good Good Average Not good Very poor

# 17- Have you eaten more than usual during the COVID-19 pandemic? *

## Mark only one oval.

Less than usual About the same More than usual

# 18- How has your diet been during the COVID-19 pandemic? *

## Mark only one oval.

Less healthy than usual

About the same healthiness as usual More healthy than usual

# 19- Rank the following activities you have spent your time on during the * COVID-19 pandemic. 19.1- Phoning or video calling with colleagues while working remotely

*Mark only one oval.*

1 2 3 4 5

e at

all


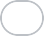

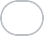

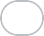

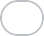

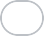


Non

A lot

# 19.2- Going to work outside of the house (for example to the oﬃce) *

*Mark only one oval.*

1 2 3 4 5

e at

all


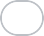

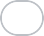

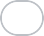

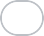

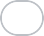


Non

A lot

# 19.3- Phoning or video calling with friends or family *

*Mark only one oval.*

1 2 3 4 5

e at

all


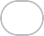

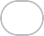

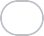

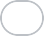

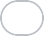


Non

A lot

# 19.4- Communicating with friends or family via email, WhatsApp, text or other *

messaging services

*Mark only one oval.*

1 2 3 4 5

e at

all


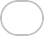

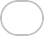

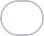

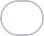

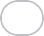


Non

A lot

# 19.5- Caring for a friend or relative *

*Mark only one oval.*

1 2 3 4 5

e at

all


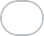

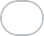

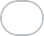

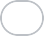

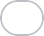


Non

A lot

# 19.6- Volunteering *

*Mark only one oval.*

1 2 3 4 5

Non A lot

e at

all

# 19.8- Going out of the house to go shopping *

*Mark only one oval.*

1 2 3 4 5

e at

all


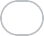

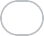

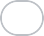

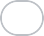

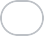


Non

A lot

# 19.9- Going out for a walk or other gentle physical activity *

*Mark only one oval.*

1 2 3 4 5

e at

all


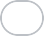

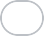

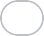

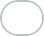

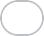


Non

A lot

# 19.10- Going out for moderate or high-intensity activity (for example, running, *

cycling or swimming)

*Mark only one oval.*

1 2 3 4 5

Non A lot

e at

all

# or indoor exercise)

*Mark only one oval.*

1 2 3 4 5

e at

all


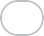

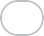

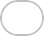

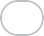

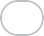


Non

A lot

# 19.12- Gardening *

*Mark only one oval.*

1 2 3 4 5

e at

all


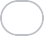

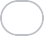

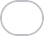

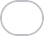

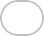


Non

A lot

# 19.13- Looking after pets *

*Mark only one oval.*

1 2 3 4 5

e at

all


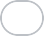

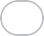

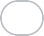

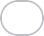

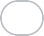


Non

A lot

# 19.14- Taking naps during the day *

*Mark only one oval.*

1 2 3 4 5

Non A lot

e at

all

# etc.)

*Mark only one oval.*

1 2 3 4 5

e at

all


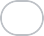

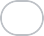

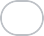

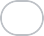

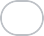


Non

A lot

# 19.16- Caring for children (for example bathing, feeding, doing homework with, *

etc.)

*Mark only one oval.*

1 2 3 4 5

e at

all


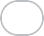

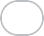

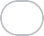

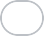

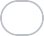


Non

A lot

# 19.17- Playing cards or board games with adults *

*Mark only one oval.*

1 2 3 4 5

e at

all


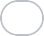

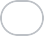

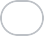

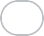

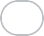


Non

A lot

# 19.18- Playing video or computer games alone, or with adults or children *

*Mark only one oval.*

1 2 3 4 5

e at

all


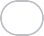

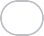

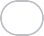

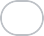

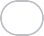


Non

A lot

# information about COVID-19

*Mark only one oval.*

1 2 3 4 5

e at

all


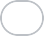

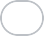

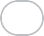

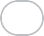

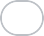


Non

A lot

# 19.20- Tweeting, blogging or posting content online about COVID-19 *

*Mark only one oval.*

1 2 3 4 5

e at

all

Non

A lot

# 19.21- Watching TV, Netﬂix, etc. (NOT for information on COVID-19) *

*Mark only one oval.*

1 2 3 4 5

e at

all

Non

A lot

# 19.22- Listening to the radio or music (NOT for information on COVID-19) *

*Mark only one oval.*

1 2 3 4 5

e at

all

Non

A lot

# 19.24- Reading *

*Mark only one oval.*

1 2 3 4 5

e at

all

Non

A lot

# 19.25- Engaging in a home-based arts or crafts activity (for example painting, *

creative writing, sewing, etc.)

*Mark only one oval.*

1 2 3 4 5

e at

all

Non

A lot

# 19.26- Engaging in a digital art activity (for example streaming a concert, *

virtual tour of a museum, etc.)

*Mark only one oval.*

1 2 3 4 5

e at

all

Non

A lot

# 19.28- Praying *

*Mark only one oval.*

1 2 3 4 5

e at

all

Non

A lot

# 19.29- Procrastinating or not doing anything in particular *

*Mark only one oval.*

1 2 3 4 5

e at

all

Non

A lot

# 20- Did you start doing new activities (something you are not used to doing) *

during the COVID-19 pandemic?

## Mark only one oval.

Yes No

# 20.1- If yes, please indicate what those new activities are?

1. 21- From where do you get most of your information about COVID-19? *

## Mark only one oval.

T.V.

Social Media

Phone calls from family friends or co-workers Text messages from officials

News from official sources Paper-based publication

# 22- What are you most looking forward to once the COVID-19 pandemic has *

ended? Please describe in 1-3 words

# 23- Did your quarantine experience during the COVID-19 pandemic so far, *

cause any life-changing decisions for you in the future or change your priorities?

Third Social Media Questions

Please answer the following questions about your social media usage during COVID- 19 (Coronavirus) pandemic.

# 1- How often currently do you go on social media (on average, across all the *

social media platforms that you use)?

## Mark only one oval.

Almost never or rarely Just about every month Every couple of days Just about every day Every couple of hours Just about every hour Every couple of minutes

# 2- On average how much time do you think you spend on social media per day? *

## Mark only one oval.

Less than 1 Hour. 1-2 Hours

2-3 Hours

3-5 Hours

5-7 Hours

More than 7 Hours

# 3- Every time you log in to social media, on average how long do you spend *

logged in (on average, across all the social media platforms that you use)?

## Mark only one oval.

About 15 minutes or less About 30 minutes

About an hour More than an hour

# 4- Do you feel it is healthy to spend that much time online? *

## Mark only one oval.

Yes No

# 5- When do you currently access social media? (Choose as many as *

applicable)

*Check all that apply.*

Morning (from 5 am to 11:59 am) Afternoon (from 12:00 pm to 6:00 pm) Evening (from 6:00 pm to 11:59 pm) Midnight (exactly 12:00 am to 4:59 am)

# 6- Do you consider yourself addicted to social media? *

## Mark only one oval.

Yes No

# 7- What do you use social media for? (Choose as many as applicable) *

*Check all that apply.*

Keeping in touch with friends and family Event planning

Buying and selling Inspiration

News about COVID-19 (Coronavirus) pandemic To make new friends

To find employment To browse/ time waste To raise awareness

To provide support to others To share your posts

To work

None of the above

# 8- Does social media distract you when you need to be productive? *

## Mark only one oval.

Yes No

# 9- Do you care about how many people like or view your posts/pictures? *

## Mark only one oval.

Yes No

# 10- Have you ever been cyberbullied in any way through social media? *

## Mark only one oval.

Yes No

# 11- When you see someone post about the intriguing events going on in their *

life, are you depressed by the idea that your life isn't as "cool" or "eventful" as theirs?

## Mark only one oval.

Yes No

# 12- When you see pictures posted of a person who has the body type that you *

desire, do you put yourself down or think negatively about your body?

## Mark only one oval.

Yes No

# 13- Do you accept friend requests/followers that you do not know in order to be *

viewed as more popular?

## Mark only one oval.

Yes No

# 14- Has social media affected your relationship with family members? *

## Mark only one oval.

Yes No

# 14.1- If yes, can you indicate the type of effect social media has on your relationship with family members?

## Mark only one oval.

Positive effect Negative effect

# 15- Do you feel as if you have an online persona? (Are you a different person *

online?)

## Mark only one oval.

Yes No

# 15.1- If yes, why do you feel you cannot be yourself? (Short answer)

1. 16- Do you feel social media has impacted your mental health? *

## Mark only one oval.

Yes No

# 16.1- If yes, in what ways has it affected you? (Choose as many as applicable)

*Check all that apply.*

Anxiety

Self-Esteem Depression

Body Dysmorphia Addiction to Social Media Eating Disorder

It has not affected me None of the above

# 17- What emotions do you experience when using social networking sites? *

(Choose as many as applicable)

*Check all that apply.*

Rejection Happiness

Boost self-esteem Jealousy Motivation Inspiration

Fear of missing out Lower self-esteem Sense of belonging None of the above

# 18- Which social media do you feel affects your mental health the most and *

why?

# 19- Personally, what do you think the positives and negatives are of social *

media?

# 20- Choose a number from 0 to 5 to the following most used social media *

platforms based on your usage where (0 is a social media platform that you do not use), (1 is the most negative social media platform in your opinion), and (5 is the most positive social media platform in your opinion): 20.1-Facebook

## Mark only one oval.

- 1. I do not use this platform
  2. Most negative social media platform in your opinion 2 Negative

1. Neutral
2. Positive
3. Most positive social media platform in your opinion

# 20.2-Instagram *

## Mark only one oval.

1. I do not use this platform
2. Most negative social media platform in your opinion 2 Negative
3. Neutral
4. Positive
5. Most positive social media platform in your opinion

# 20.3-LinkedIn *

## Mark only one oval.

1. I do not use this platform
2. Most negative social media platform in your opinion 2 Negative
3. Neutral
4. Positive
5. Most positive social media platform in your opinion

# 20.4-Snapchat *

## Mark only one oval.

1. I do not use this platform
2. Most negative social media platform in your opinion 2 Negative
3. Neutral
4. Positive
5. Most positive social media platform in your opinion

# 20.5-Telegram *

## Mark only one oval.

1. I do not use this platform
2. Most negative social media platform in your opinion 2 Negative
3. Neutral
4. Positive
5. Most positive social media platform in your opinion

# 20.6-TikTok *

## Mark only one oval.

1. I do not use this platform
2. Most negative social media platform in your opinion 2 Negative
3. Neutral
4. Positive
5. Most positive social media platform in your opinion

# 20.8-WhatsApp *

## Mark only one oval.

1. I do not use this platform
2. Most negative social media platform in your opinion 2 Negative
3. Neutral
4. Positive
5. Most positive social media platform in your opinion

# 20.9-YouTube *

## Mark only one oval.

1. I do not use this platform
2. Most negative social media platform in your opinion 2 Negative
3. Neutral
4. Positive
5. Most positive social media platform in your opinion

# 21- If there is a social media platform or platforms you would like to add to the previous list please add it below, indicate whether you think it is positive or negative, and mention why?

1. 22- In all of the questions, you have answered in this part about your social *

# media usage how different is your social media usage to how it was before the pandemic?

## Mark only one oval.

Much worse A little worse No different A little better Much better

Fourth Mental Health Questions

Please answer the following questions about your mental health during COVID-19 (Coronavirus) pandemic.

Better than usual Same as usual Less than usual

Much less than usual

# 2- Lost much sleep over worry? *

## Mark only one oval.

Not at all

No more than usual Rather more than usual Much more than usual

# 3- Felt that you are playing a useful part in things? *

## Mark only one oval.

More so than usual Same as usual Less so than usual

Much less than usual

# 4- Felt capable of making decisions about things? *

1. 6- Felt you couldn’t overcome your diﬃculties? *

## Mark only one oval.

Not at all

No more than usual Rather more than usual Much more than usual

# 7- Been able to enjoy your normal day to day activities? *

## Mark only one oval.

More so than usual Same as usual Less so than usual

Much less than usual

# 8- Been able to face up to your problems? *

1. 9- Been feeling unhappy or depressed? *

## Mark only one oval.

Not at all

No more than usual Rather more than usual Much more than usual

# 10- Been losing conﬁdence in yourself? *

## Mark only one oval.

Not at all

No more than usual Rather more than usual Much more than usual

# 11- Been thinking of yourself as a worthless person? *

## Mark only one oval.

Not at all

No more than usual Rather more than usual Much more than usual

# 12- Been feeling reasonably happy, all things considered? *

## Mark only one oval.

More so than usual Same as usual Less so than usual

Much less than usual

# 13- In all of the questions, you have answered in this part about your mental *

health how different are these feelings about your mental health to how you felt before the pandemic?

## Mark only one oval.

Much worse A little worse No different A little better Much better

For more information about mental health support, you can refer to <https://www.mind.org.uk/>or <https://www.samaritans.org/>

Fifth Family Functioning Questions

# Please answer the following questions about your family functioning during COVID-19 (Coronavirus) pandemic.

1. 1- Planning family activities is diﬃcult because we misunderstand each other *

## Mark only one oval.

Strongly agree Agree Disagree

Strongly disagree

# 2- In the time of crisis, we can turn to each other for support as a family *

## Mark only one oval.

Strongly agree Agree Disagree

Strongly disagree

# 3- As a family, we cannot talk to each other about the sadness we feel *

## Mark only one oval.

Strongly agree Agree Disagree

Strongly disagree

# 4- In my family, individuals are accepted for what they are *

## Mark only one oval.

Strongly agree Agree Disagree

Strongly disagree

# 5- We avoid discussing our fears and concerns as a family *

1. 7- There are lots of bad feelings in my family *

## Mark only one oval.

Strongly agree Agree Disagree

Strongly disagree

# 8- In my family, I feel accepted for what I am *

## Mark only one oval.

Strongly agree Agree Disagree

Strongly disagree

# 9- Making decisions is a problem for my family *

1. 11- My family do not get along well together *

## Mark only one oval.

Strongly agree Agree Disagree

Strongly disagree

# 12- As a family, we conﬁde in each other *

## Mark only one oval.

Strongly agree Agree Disagree

Strongly disagree

# 13- In all of the questions, you have answered in this part about your family * functioning how different are these feelings about your family functioning to how you felt before the pandemic?

## Mark only one oval.

Much worse A little worse No different A little better Much better

For more information about family support, you can refer to <https://www.familylives.org.uk/how-we-can-help/>or <https://www.familysupportni.gov.uk/>

This content is neither created nor endorsed by Google.

[Forms](https://www.google.com/forms/about/?utm_source=product&utm_medium=forms_logo&utm_campaign=forms)
