# Supplementary material for: The Impact of Social Media Use on Mental Health and Family Functioning Within Web-Based Communities in Saudi Arabia: Ethnographic Correlational Study
Source: JMIR Form Res. 2024 Jan 16;8:e44923. doi: 10.2196/44923 (PMC10828947; doi:10.2196/44923)
Supplement: Multimedia Appendix 2 [file formative_v8i1e44923_app2.docx]

# Statistical analysis tables for the paper titled (An Ethnographic Study on the Impact of Social Media Usage on Mental Health and Family Functioning on Saudi Arabia Online Communities)

Table S1. Demographics of participants.

|  | **N** | **%** |
| --- | --- | --- |
| **Age** |  |  |
| 18-24 years old | 34 | 10.8% |
| 25-34 years old | 57 | 18.2% |
| 35-44 years old | 80 | 25.5% |
| 45-54 years old | 58 | 18.5% |
| 55-64 years old | 75 | 23.9% |
| 65-74 years old | 10 | 3.2% |
| **Gender** |  |  |
| Male | 76 | 24.2% |
| Female | 234 | 74.5% |
| Prefer not to say | 4 | 1.3% |
| **Residency** |  |  |
| In Saudi Arabia | 293 | 93.3% |
| Outside Saudi Arabia | 21 | 6.7% |
| **Suffering from psychological condition** |  |  |
| No | 271 | 86.3% |
| Yes | 9 | 2.9% |
| Not sure | 34 | 10.8% |
| **Suffering from medical condition** |  |  |
| No | 216 | 68.8% |
| Yes | 83 | 26.4% |
| Not sure | 15 | 4.8% |
| **Race** |  |  |
| Arab | 284 | 90.4% |
| Non-Arab | 30 | 9.6% |
| **Type of place of living** |  |  |
| Villa or mansion | 105 | 33.4% |
| House in a compound | 26 | 8.3% |
| House with garden or backyard | 40 | 12.7% |
| House without garden or backyard | 26 | 8.3% |
| Flat in a flat block | 111 | 35.4% |
| Room(s) in a shared house | 3 | 1.0% |
| None of the above | 3 | 1.0% |
| **Level of Education** |  |  |
| High School | 26 | 8.3% |
| Bachelor’s degree | 138 | 43.9% |
| Master’s degree | 60 | 19.1% |
| Doctorate degree | 81 | 25.8% |
| None of the above | 9 | 2.9% |

Table S2. Perceived impact of social media platforms on mental health.

|  | N | % | Mean score |
| --- | --- | --- | --- |
| *Which social media do you feel affects your*  *mental health the most?* | | | |
| *1-Facebook* |  | | 3.25 |
| *I do not use this platform* | 157 | 50.0% |  |
| *Most negative social media platform in your opinion* | 11 | 3.5% |  |
| *Negative* | 5 | 1.6% |  |
| *Neutral* | 89 | 28.3% |  |
| *Positive* | 37 | 11.8% |  |
| *Most positive social media platform in your opinion* | 15 | 4.8% |  |
| *2- Instagram* |  | | 3.40 |
| *I do not use this platform* | 67 | 21.3% |  |
| *Most negative social media platform in your opinion* | 4 | 1.3% |  |
| *Negative* | 14 | 4.5% |  |
| *Neutral* | 128 | 40.8% |  |
| *Positive* | 82 | 26.1% |  |
| *Most positive social media platform in your opinion* | 19 | 6.1% |  |
| *3-LinkedIn* |  | | 3.47 |
| *I do not use this platform* | 237 | 75.5% |  |
| *Most negative social media platform in your opinion* | 1 | 0.3% |  |
| *Negative* | 5 | 1.6% |  |
| *Neutral* | 38 | 12.1% |  |
| *Positive* | 23 | 7.3% |  |
| *Most positive social media platform in your opinion* | 10 | 3.2% |  |
| *4-Snapchat* |  | | 3.02 |
| *I do not use this platform* | 67 | 21.3% |  |
| *Most negative social media platform in your opinion* | 27 | 8.6% |  |
| *Negative* | 47 | 15.0% |  |
| *Neutral* | 86 | 27.4% |  |
| *Positive* | 67 | 21.3% |  |
| *Most positive social media platform in your opinion* | 20 | 6.4% |  |
| *5-Telegram* |  | | 3.86 |
| *I do not use this platform* | 114 | 36.3% |  |
| *Most negative social media platform in your opinion* | 4 | 1.3% |  |
| *Negative* | 4 | 1.3% |  |
| *Neutral* | 51 | 16.2% |  |
| *Positive* | 99 | 31.5% |  |
| *Most positive social media platform in your opinion* | 42 | 13.4% |  |
| *6-Tik tok* |  | | 1.98 |
| *I do not use this platform* | 190 | 60.5% |  |
| *Most negative social media platform in your opinion* | 52 | 16.6% |  |
| *Negative* | 33 | 10.5% |  |
| *Neutral* | 31 | 9.9% |  |
| *Positive* | 6 | 1.9% |  |
| *Most positive social media platform in your opinion* | 2 | 0.6% |  |
| *7-Twitter* |  | | 3.62 |
| *I do not use this platform* | 59 | 18.8% |  |
| *Most negative social media platform in your opinion* | 11 | 3.5% |  |
| *Negative* | 15 | 4.8% |  |
| *Neutral* | 83 | 26.4% |  |
| *Positive* | 96 | 30.6% |  |
| *Most positive social media platform in your opinion* | 50 | 15.9% |  |
| *8-WhatsApp* |  | | 4.08 |
| *Most negative social media platform in your opinion* | 5 | 1.6% |  |
| *Negative* | 8 | 2.5% |  |
| *Neutral* | 60 | 19.1% |  |
| *Positive* | 124 | 39.5% |  |
| *Most positive social media platform in your opinion* | 117 | 37.3% |  |
| *9-YouTube* |  | | 3.68 |
| *I do not use this platform* | 9 | 2.9% |  |
| *Most negative social media platform in your opinion* | 1 | 0.3% |  |
| *Negative* | 10 | 3.2% |  |
| *Neutral* | 118 | 37.6% |  |
| *Positive* | 132 | 42.0% |  |
| *Most positive social media platform in your opinion* | 44 | 14.0% |  |
| *10-Pinterest* |  | | 3.85 |
| *I do not use this platform* | 217 | 69.1% |  |
| *Most negative social media platform in your opinion* | 1 | 0.3% |  |
| *Negative* | 3 | 1.0% |  |
| *Neutral* | 29 | 9.2% |  |
| *Positive* | 41 | 13.1% |  |
| *Most positive social media platform in your opinion* | 23 | 7.3% |  |

Table S3. Social media usage habits and their impact.

|  | *N* | *%* |
| --- | --- | --- |
| *Do social media distract you when you need to be productive?* |  |  |
| *No* | 208 | 66.2% |
| *Yes* | 106 | 33.8% |
| *Do you care about how many people like or view your posts/pictures?* |  |  |
| *No* | 237 | 75.5% |
| *Yes* | 77 | 24.5% |
| *Have you ever been cyber bullied in any way through social media?* |  |  |
| *No* | 276 | 87.9% |
| *Yes* | 38 | 12.1% |
| *When you see someone post about the intriguing events going on in their life, are you depressed by the idea that your life isn't as "cool “as theirs?* |  |  |
| *No* | 269 | 85.7% |
| *Yes* | 45 | 14.3% |
| *When you see pictures posted of a person who has the body type that you desire, do you put yourself down or think negatively about your body?* |  |  |
| *No* | 217 | 69.1% |
| *Yes* | 97 | 30.9% |
| *Do you accept friend requests/followers that you do not know in order to be viewed as more popular?* |  |  |
| *No* | 266 | 84.7% |
| *Yes* | 48 | 15.3% |
| *Has social media affected your relationship with family members?* |  |  |
| *No* | 241 | 76.8% |
| *Yes* | 73 | 23.2% |
| *If yes, can you indicate the type of effect social media has on your relationship with family members?* |  |  |
| *Positive effect* | 48 | 15.3% |
| *Positive effect* | 50 | 15.9% |
| *Do you feel as if you have an online persona?* |  |  |
| *No* | 286 | 91.1% |
| *Yes* | 28 | 8.9% |
| *Do you feel social media has impacted your mental health?* |  |  |
| *No* | 259 | 82.5% |
| *Yes* | 55 | 17.5% |
| *If yes, in what ways has it affected you?* |  |  |
| *Anxiety* | 38 | 69.09% |
| *Self-Esteem* | 17 | 30.91% |
| *Depression* | 19 | 34.55% |
| *Body dysmorphia* | 12 | 21.82% |
| *Addiction to Social Media* | 36 | 65.45% |
| *Eating Disorder* | 8 | 14.55% |
| *It has not affected me* | 14 | 25.45% |
| *None of the above* | 22 | 40.00% |
| *What emotions do you experience when using social networking sites?* |  |  |
| *Rejection* | 9 | 2.87% |
| *Happiness* | 78 | 24.84% |
| *Boost self-esteem* | 64 | 20.38% |
| *Jealousy* | 11 | 3.50% |
| *Motivation* | 83 | 26.43% |
| *Inspiration* | 159 | 50.64% |
| *Fear of missing out* | 31 | 9.87% |
| *Lower self-esteem* | 8 | 2.55% |
| *Sense of belonging* | 33 | 10.51% |
| *None of the above* | 79 | 25.16% |
